# Urine Biochemical Parameters in Predicting Severity of SARS-CoV-2 Infection: an Experience in Tertiary Care Centre in Western India

**DOI:** 10.30699/IJP.2021.136576.2496

**Published:** 2021-06-12

**Authors:** Priyanka Murgod, Preeti Doshi, Ravindra Nimbargi

**Affiliations:** 1 *Department of Pathology, Maharashtra Institute of Medical Education and Research (MIMER), Talegaon Dabhade, Pune, India*; 2 *Department of Pathology, Bharati Vidyapeeth (DTU) Medical College and Hospital, Pune, India*

**Keywords:** Blood, Protein, SARS-COV-2, Urinary biochemical parameters

## Abstract

**Background & Objective:**

Coronavirus is an enveloped RNA virus that mainly causes respiratory infection. Real-time reverse transcriptase polymerase chain reaction (RT-PCR) test of nasopharyngeal and oropharyngeal swab is the confirmatory diagnostic test for severe acute respiratory syndrome coronavirus 2 (SARS-COV-2) infection. The relationship between SARS-COV-2 and body fluid parameters is still not known. There have been few studies regarding the correlation between urine biochemical parameters and SARS-COV-2 infection. The aim of the study is to determine the importance of urinary biochemical parameters in SARS-COV-2 infection and whether these parameters can be used to predict the severity of the infection.

**Methods:**

This was a retrospective observational study consisting of total of 285 patients diagnosed with SARS-COV-2 infection. The patients were divided into three groups according to the severity of infection as mild (120 cases), moderate (110 cases) and severe (55 cases). During the study period 72 healthy persons were enrolled as controls. Analysis was done to find any relationship between various urine biochemical parameters and the severity of SARS-COV-2 infection.

**Results:**

Urinary occult blood (U. Blood) and Urinary protein (U. Pro) have higher positive rates in SARS-COV-2 patients as compared with healthy controls. Among the severities of SARS-COV-2 infection (mild, moderate and severe), both these parameters were significantly higher. Glucose (Glu) and Ketone (Ket) positivity rate was more in moderate cases of SARS-COV-2 than mild cases.

**Conclusion:**

Urinary biochemical parameters are very useful in identification of SARS-COV-2 infection and also have the advantage in evaluating the progression in patients infected with SARS-COV-2. Among the different parameters, Urinary Occult Blood and Urinary protein are significant in the differentiation of SARS-COV-2 severity.

## Introduction

Coronavirus is an RNA virus which causes mainly respiratory infection in humans. (1) After its first discovery in China, Wuhan, the coronavirus illness known by the name, severe acute respiratory syndrome coronavirus 2 (SARS-COV-2), spread throughout the world and the World Health Organization (WHO) declared it as pandemic (2). The pandemic affected many countries with more than 2.5 million people infected and causing thousands of deaths. Many countries are faced with the burden of overwhelmed healthcare systems. Additionally, due to limited diagnostic tests availability, an under-reporting of cases is happening (3-5). SARS-CoV-2 virus presents as acute respiratory illness and involves multiple organs (6, 7). Many studies have established that the involvement of urinary system is common in patients infected with SARS-COV-2 with progressive deterioration of kidney function as an unfavorable prognostic factor (8). Urine dipstick tests are quick, economical and noninvasive. The different chemical parameters of dipstick test are used to diagnose urinary tract infections (UTIs), renal infections and to monitor various treatment effects (1, 9). There are a few studies with correlation between urine biochemical parameters and SARS-COV-2. The aim of this study was to determine the importance of urinary biochemical parameters in SARS-COV-2 infection and whether these parameters can be used to predict the severity of the infection.

## Materials and Methods

A retrospective analytical study was done for one month, June 2020. A total of 285 patients diagnosed with SARS-COV-2 infection, representing as case group were included in this study along with 72 healthy subjects as control group. The case group was further subdivided into three groups, according to the National Clinical Management Protocol: SARS-COV-2 infection by Government of India. 

**The case groups are as follows:**

1.** Mild group**- Patients with pneumonia and no signs of severe pneumonia; 

2. **Moderate -** fever or suspected respiratory infection, plus any of the following like; respiratory rate >30 breaths/min, severe respiratory distress, SpO_2_ <90%; 3.** Severe**- new or worsening respiratory symptoms within one week of known clinical or chest imaging showing bilateral opacities, not fully explained by effusions, lobar or lung collapse, or nodules. 

The infection diagnosis criteria were based on current standards, positive result on oropharyngeal and nasopharyngeal swabs by RT-PCR for SARS-CoV-2 infection. Patients with underlying chronic diseases like Hypertension, diabetes mellitus, chronic kidney disease and asymptomatic patients (home isolation) were excluded from the study.

After diagnosis of SARS-CoV-2 infection, 20 to 30 mL of clean midstream urine samples were collected from patients in each subgroup mentioned above. Urine samples were collected from catheter in critical patients. All biochemical parameters of urine urobilinogen (UBG), bilirubin, ketone (Ket), urinary occult blood (U. Blood), urinary protein (U. Pro), Urinary Glucose (U. Glu), nitrite (Nit), leukocyte esterase (LeuE), Potential of hydrogen (pH) and specific gravity (SG) were analyzed using urine biochemical analyzer (Uriplus 600, China) and multistick uristrips (Aspen Diagnostics, India). The collected samples were processed within 2 hours. Refrigeration of collected samples was done at 4 to 6ºC in case of delay. For suspicious results, the tests were confirmed manually whenever possible.

**Statistical Analysis**

The statistical software STAT15 was used for analysis. Chi-square (χ2) test was used to analyze the data. Mean ± SD was done for normally distributed data and it was analyzed using t-test. The data was statistically significant if P-value was <0.05.

## Results

Overall, males accounted for 185 SARS-COV-2 infected cases (median age, 47.38 years in mild cases, 57 years in moderate cases and 60.6 years in severe cases) and females accounted for 100 cases (median age, 42.5 years in mild cases, 58.6 years in moderate cases and 9.1 years in severe cases). Moderate and severe cases of SARS-COV-2 infection were seen maximum in 5^th^ and 6^th^ decade of life while the ratio of male to female preponderance was 1.3:1 as shown in Table 1. 

**Table 1:** Comparison of mean age, sex and number of cases among case group and the control group

Group	Gender	Age (years)	Number
Healthy	Female	40 (23-57)	20
	Male	39 (23-55)	52
	Female	42(25-59)	39
Mild	Male	47 (34-60)	81
	Female	58 (43-73)	45
Moderate	Male	57(44-70)	65
	Female	49 (36-62)	16
Severe	Male	60 ± 13	39
		x^2^=3.8290	x^2^=4.2934
		*P*=0.04	*P*=0.231

Table 2 shows that among the urine biochemical parameters, the differences in Urinary Blood and Urinary Protein values were statistically significant (p < 0.05) in patients with SARS-COV-2 infection and healthy controls. Blood in SARS-COV-2 patients was found to be 17.5% while in control group was 5.6% and Pro in SARS-COV-2 was 78.2 % and in control group was 34.7%. Other parameters like pH, SG, Leu and Glu were found to be statistically insignificant.

Among the different subgroups of SARS-COV-2 patients of mild, moderate and severe cases, the positivity rate of Blood in mild is 5%, moderate 20.9% and severe 38.2%. It is found to be significantly higher in moderate and severe cases as compared to mild cases. The positivity rate of Pro is 68.3% in mild, 87.3% in moderate and 81.8% in severe cases. The maximum positivity rates of Glu and Ket was seen higher in individuals with moderate SARS-COV-2 infection than in those with mild and severe SARS-COV-2. Among the different severities of SARS-COV-2, the differences in the positivity rates of leucocyte esterase and nitrite were not statistically significant (Table 3).

**Table 2:** Comparison of urine biochemical parameters among patients and controls

	N	Urinary Occult Blood	Leukocyte esterase	Urinary Protein	Specific Gravity (SG)	Potential of hydrogen (pH)
SARS-COV-2	285	50 (17.5%)	7 (2.5%)	223 (78.2%)	1.023 ± 0.006	5.8 ± 0.77
Healthy	72	4 (5.6%)	4 (5.6%)	25 (34.7%)	1.022 ± 0.008	5.9 ± 0.75
x2/t		30.1075	1.849	51.3349	1.5038	1.1152
P-value		<0.05	0.174	<0.05	0.1335	0.2655

**Table 3:** Evaluation of the severity of SARS-COV-2 cases by urine biochemical parameters among different subgroups (mild, moderate and severe cases)

	Mild (n = 120)	Moderate (n = 110)	Severe (n = 55)	x^2^/t	P-value
U.Blood	6 (5%)	23 (20.9%)	21 (38.2%)	30.1075	<0.05
U. Glu	34 (28.3%)	42 (38.2%)	13 (23.6%)	4.4209	0.11
Ketone	6 (5%)	22 (20%)	7 (12.7%)	2.5995	0.273
LeuE	3 (2.5%)	3 (2.7%)	1 (1.8%)	0.1281	0.938
U. Pro	82 (68.3%)	96 (87.3%)	45 (81.8%)	12.6051	<0.05

- urinary occult blood (U. Blood), -leukocyte esterase (LeuE), -urinary protein (U. Pro), -Urinary glucose (U. Glu)

## Discussion

Novel Coronavirus, SARS-COV-2 infection out broke and spread to the world in 2019. On 11^th^ march 2020 SARS-COV-2 infection was declared as pandemic by World Health Organization (WHO). SARS-COV-2 infection has been impacting a large number of people worldwide, being reported in approximately 215 countries and territories (7, 10, 11). An RNA-based metagenomic next-generation sequencing (NGS) approach detected SARS-CoV-2 virus as the cause of this infection (1, 12, 13, 14). Having a strong binding affinity to the angiotensin converting enzyme 2 (ACE 2) receptors, Coronavirus binds to the receptor and enters the host cells to cause infection (15). These ACE 2 receptors mainly exist on Type II alveolar epithelial cells and are also expressed in multiple other organs like kidney, heart, gut cells and endothelium (16). Hence, many studies report involve-ment of multiple organs including kidney by this virus. Clinical features of SARS-COV-2 infection are variable and ranges from asymptomatic state with mild or no symptoms to critical stage causing acute respiratory distress with sepsis and multi organ failure (17, 18). During initial outbreak of the infection, urine samples were not routinely collected in infected patients in many countries as there was delay in properly diagnosing SARS-COV-2 infection (6, 19). Later, many publications and studies highlighted the rarity of SARS-COV-2 viral infection in urine. Also, recently several studies demonstrated the presence of SARS-COV-2 virus in urine (20, 21).

Few publications have highlighted a positive association between analysis of urine sample and progression of the illness (1, 8). In our study, it is been observed that urine proteins and urine occult blood were positive and higher in SARS-COV-2 patients as compared to healthy controls. But, the positivity rate of leucocyte esterase was not statistically significant between SARS-CoV-2 patients and healthy controls. This indicates that the difference in positivity rates of urine occult blood and urinary protein are due to SARS-CoV-2 infection and not because of bacterial infection. An interesting observation was also that urinary glucose and ketone positivity rates were higher in moderate cases of SARS-COV-2 than mild cases. The positivity rates of urinary occult blood and urinary protein were also significantly different among the mild, moderate and severe cases of the viral infection. The positivity rate of both these parameters was significantly higher in moderate and severe cases when compared to mild cases which are concordant with other studies (1, 5).

The study done by Liu *et al.* (2020) found that the urine occult blood and urinary protein positivity rates were higher in patients infected with SARS-COV-2 when compared with healthy group which is concordant with our study (1). Liu *et al.* also observed that SG and pH was statistically significant, with P<0.05 and was higher in SARS-COV-2 patients as compared with healthy controls which. Bonetti *et al.* (5, 22) found similar findings of proteinuria and hematuria in SARS-COV-2 patients.

In infection mediated multiple organ failure syndromes, cytokine storm plays a major role. Cytokine storm syndrome developing secondary to SARS-COV-2 infection can indirectly cause cellular damage in the kidneys. Also, the virus can directly enter the urinary tract by binding to the ACE2 receptors and thereby causing renal dysfunction (1, 6, 23-26). Thus this renal dysfunction could be accountable for positivity rates of urine occult blood and urinary protein parameters in the subgroups of mild, moderate and severe cases of infection. It is also observed that as kidney damage progresses, both these parameters are found to be significantly higher in moderate and severe cases. Another important observation noted in the study was that involvement of kidney can be used as a predictor of unfavorable disease progression. Thus, this emphasizes the importance of routine urine analysis for risk stratification of SARS-COV-2 infected patients (28-30). It was also noted that the positivity rate of ketone was not significantly different among the different subgroups of SARS-COV-2 patients, indirectly confirming that the shock developing in patients is because of impaired lung function and not due to ketoacidosis. 

Our study has made the observation that few urine chemical parameters, especially urine occult blood and urinary protein are significantly higher in SARS-CoV-2 infection as compared with controls. Also both these parameters can be used for differentiating mild, moderate and severe cases and predicting the course of the infection.

 The limitation of our study was that there was a large number of asymptomatic patients with SARS-COV-2 infection and these patients were rarely admitted in hospitals and were mostly referred to temporary/home isolation for treatment. So it was difficult to obtain information or data from these patients.

## Conclusion

Urine biochemical parameters can be used in the diagnosis and also in the evaluation of progression in patients with SARS-COV-2 infection. We hence concluded that routine urinalysis should be performed in all SARS-COV-2 infected patients as it also provides significant biochemical information for clinical management.
